# Case Report: Preimplantation Genetic Testing for X-Linked Severe Combined Immune Deficiency Caused by *IL2RG* Gene Variant

**DOI:** 10.3389/fgene.2022.926060

**Published:** 2022-06-01

**Authors:** Jun Ren, Cuiting Peng, Fan Zhou, Yutong Li, Yuezhi Keqie, Han Chen, Hongmei Zhu, Xinlian Chen, Shanling Liu

**Affiliations:** ^1^ Department of Medical Genetics, Center of Prenatal Diagnosis, West China Second University Hospital, Sichuan University, Chengdu, China; ^2^ Department of Obstetrics and Gynecology, West China Second University Hospital, Sichuan University, Chengdu, China; ^3^ Key Laboratory of Birth Defects and Related Diseases of Women and Children (Sichuan University), Ministry of Education, Chengdu, China

**Keywords:** preimplantation genetic testing, severe combined immune deficiency, IR2RG, haplotype, rare genetic disease, next generation sequencing

## Abstract

Preimplantation genetic testing (PGT) has been increasingly used to prevent rare inherited diseases. In this study, we report a case where PGT was used to prevent the transmission of disease-caused variant in a SCID-X1 (OMIM:300400) family. SCID-X1 is an X-linked recessive inherited disease whose major clinical manifestation of immune deficiency is the significant reduction in the number of T-cells and natural killer cells. This family gave birth to a boy who was a hemizygous proband whose *IL2RG* gene was mutated (c.315T > A, p(Tyr105*), NM_000206.3, CM962677). In this case, Sanger sequencing for mutated allele and linkage analysis based on single-nucleotide polymorphism (SNP) haplotype via next-generation sequencing were performed simultaneously. After PGT for monogenic disorder, we detected the aneuploidy and copy number variation (CNV) for normal and female carrier embryos. Four embryos (E02, E09, E10, and E11) were confirmed without CNVs and inherited variants at the *IL2RG* gene. Embryo E02 (ranking 4BB) has been transferred after considering the embryo growth rate, morphology, and PGT results. Prenatal genetic diagnosis was used to detect amniotic fluid cells, showing that this fetus did not carry the variant of the *IL2RG* gene (c.315T > A). Ultimately, a healthy girl who had not carried disease-causing variants of SCID-X1 confirmed by prenatal diagnosis was born, further verifying our successful application of PGT in preventing mutated allele transmission for this SCID family.

## Introduction

Severe combined immune deficiency (SCID, OMIM:300400) is a fatal genetic defect (Torii, 1996). Although SCID morbidity varies from country to country, there is no report on its exact incidence in China. According to research on newborn screening in the United States, SCID affects one in 58,000 infants (95% CI, 1/46000–1/80000) (Kwan et al., 2014). SCID is a prenatal disorder of T lymphocyte development (Cossu, 2010), in which the affected infant gradually develops a pediatric emergency after birth. In general, the affected infant presents severe opportunistic infections within 1 month of birth because of defects in humoral and cellular immunity (Mamcarz et al., 2019). Typical laboratory inspections show a lack of T cells, natural killer (NK) cells, and functional B cells. As a result, affected infants cannot usually live beyond their first year of life.

The most common cause of SCID is a variant of the *IL2RG* gene (OMIM: 300400), also called SCID-X1. The *IL2RG* gene, situated in Xq13.1, encodes the interleukin-2 receptor common gamma chain and is shared by several cytokine receptors necessary for the development and function of lymphocytes (Mamcarz et al., 2019). Allogeneic hematopoietic stem-cell transplantation or autologous gene therapy is considered the most effective treatment for SCID (Cavazzana-Calvo et al., 2000). Although hematopoietic stem cell transplantation (HSCT) from a matched sibling donor is effective, it can only be used in a minority of patients, as transplantation from an alternative donor is related to an increased risk of graft-versus-host disease and incomplete immune reconstitution (Mamcarz et al., 2019). Gene therapy has also shown potential in this regard, but the carcinogenicity of retroviral vectors remains to be solved. Leukemia was caused when gene therapy was first used to treat SCID-X1, owing to an insertional variant induced by enhancers of the adenovirus vectors (Fischer and Hacein-Bey-Abina, 2020). With the development of gene therapy vectors, such as lentivirus, increasingly exciting research has been carried out to prove their application value in SCID therapy (Blanco et al., 2020).

However, for these couples with an inherited disease that can be clearly diagnosed genetically, adequate genetic counseling and prenatal or preimplantation genetic diagnostic techniques are important (De Rycke and Berckmoes, 2020, Group et al., 2020). With the development of assisted reproduction and molecular genetic technology, preimplantation genetic testing (PGT) has been used in birth defect prevention and control (Group et al., 2020). PGT for monogenic disorders (PGT-M) can select unaffected embryos to prevent the transmission of disease-causing variants. PGT technology avoids the adverse effects of repeated abortion on women’s physical and mental health.

Through this case, we report a PGT-M case based on MALBAC and next-generation sequencing (NGS)-based single-nucleotide polymorphism (SNP) haplotype for SCID. To further verify our PGT results, chromosome microarray (CMA) for copy number variation (CNV) analysis and Sanger sequencing for mutated alleles were conducted in amniotic cells for prenatal genetic diagnosis.

## Patients and Methods

### Patients

A 31-year-old couple had given birth to a boy who was the proband. The female carrying *IL2RG* mutated allele visited the Department of Medical Genetics, West China Second University Hospital, Sichuan University. The boy was diagnosed with X-linked SCID (OMIM:300400) in the West China Second University Hospital for repeated high fever, severe anemia, hepatosplenomegaly, immune system deficiency, coagulation dysfunction, hemangioma, and severe sepsis. The boy died at less than 1 year of age. We found that the boy had a variant in the *IL2RG* gene (c.315T > A, p(Tyr105*), NM_000206.3, CM962677). According to the standards and guidelines for sequence variant interpretation of the American College of Medical Genetics and Genomics (ACMG/AMP) (Richards et al., 2015), Clinical Interpretation of Sequence Variants (zhang et al., 2020) and ClinGen Sequence Variant Interpretation Recommendation for PM2 - Version 1.0, we evaluated this variant with PVS1 + PM2_Supporting, likely pathogenic variant. Parents received genetic counseling and signed an informed consent form. Sample collection, library preparation, NGS and data analysis were conducted at the Department of Medical Genetics, West China Second University Hospital. This study was approved by the Internal Ethical Committees of the West China Second University Hospital.

### Assisted Reproductive Technology Procedure and Embryo Trophectoderm Biopsy

Controlled ovarian stimulation, intracytoplasmic sperm injection (ICSI), blastocyst culture, trophectoderm biopsy, and blastocyst transfer were conducted in the Reproductive Medicine Center of West China Second University Hospital, according to the standard protocol (McArthur et al., 2005; Schoolcraft et al., 2011). In this cycle, twelve embryos were finally developed into blastocysts, and the trophectoderm (TE) cells were biopsied on day 5 or 6 after insemination. A total of five to eight biopsied cells from the TE were transferred into 4.5 μL lysis buffer (Yikon Genomics) in 0.2 μL PCR tubes for whole-genome amplification (WGA) (MALBAC).

### gDNA Extraction and WGA

Peripheral blood of the couple was collected for gDNA extraction by a DNeasy Blood and Tissue Kit (Qiagen). Although the proband (hemizygote) was deceased, the couple kept his gDNA sample. In the second trimester, amniotic cells were collected by transabdominal amniocentesis; gDNA extraction was also carried out using a DNeasy Blood and Tissue Kit (Qiagen). The buccal mucosa cells (BMCs) of the woman (variant carrier) were diluted by phosphate buffer saline; five to eight cells were selected for WGA. WGA involves multiple annealing and looping-based amplification cycles (MALBAC, Yikon genomics) applied for diluted mucosa and TE cells. Additionally, 5 μL WGA products were used for 1% agarose gel electrophoresis; a 300–2000 bp diffused band indicated successful amplification. Whole-genome products were purified using a DNA Clean-up Kit (CWBIO). All operations were performed according to manufacturers’ protocols.

### Variant Site Detection

PCR amplification and Sanger sequencing were conducted to validate the mutated *IL2RG* allele. Primer was designed to amplify the segment containing c.315 of *IL2RG*. Forward primer (CTCCCAG GTACCCCACTGTT) and reverse primer (TCC​AAT​GTC​CCA​CAG​TAT​CCC) were designed (Primer 5.0 software) and synthesized (TsingKe Biotechnology, Beijing). PCR was performed in a 25 μL system using 2× GoldStar Best Master Mix (CWBIO) on a 96-Well Thermal Cycler Veriti Dx (Life Technologies). The amplification system contained 2 μL of primer mix, 12.5 μL of enzyme mix, gDNA, or purified WGA products as templates. The reaction condition was as follows: 95°C for 5 min; 95°C for 30 s, 57°C for 30 s, 72°C for 40 s (35 cycles); 72°C for 5 min; held at 4°C. Sanger sequencing data were analyzed by ChromasPro software.

Before the scheme could be used for the TE cells, we used the wife’s BMCs to imitate biopsied trophectoderm (TE) cells because the proband had passed away. The purpose is to test the effectiveness of the primers and the allele drop-out (ADO) rate of the WGA (WGA) method. MALBAC was applied for five to eight BMCs. WGA production of BMCs was used to amplify the target segments and Sanger sequencing.

### Library Preparation and NGS

Purified WGA products were used for the PCR and SNP-based haplotype via NGS. An NGS library preparation kit (Yikon Genomics) was used to prepare the SNP library. For the CNV library, unpurified WGA products were used via an NGS library preparation kit (Yikon Genomics). All operations followed the manufacturers’ protocols. Library sequencing was performed in the MiSeq Dx platform using a MiSeq Reagent Kit v3 (150-cycles) (Illumina). The raw data were automatically filtered, generating FASTQ files; the Q30 should be greater than 90%.

### CNV Analysis and NGS-Based SNP Haplotype

For CNV analysis, FASTQ files were disposed of in the local analysis platform ChromGo (Yikon Genomics). More than 4 Mb deletions or duplications were reported. For CNV analysis, valid reads should be more than 1 Mb, CV (1000K_bin_size), and valid read GC contents should be in an acceptable range. To detect ADO and recombination, an NGS-based haplotype was conducted using SNP within the 2-million base pair (Mb) region, flanking the targeted gene. In this case, 60 SNPs were selected. For the SNP haplotype, bioinformatics analysis was conducted by Yikon Genomics, Ltd. The informative SNP sites are homozygous in the spouse and heterozygous in the variant carrier. Proband haplotypes were used for reference.

### Embryo Transplantation and Prenatal Genetic Diagnosis

For PGT-M, embryo selection involves comprehensive considerations (Munné et al., 2019), including quality, the developmental stage of the embryo, and the results of PGT-A&M. Results were confirmed by the prenatal genetic diagnosis of amniotic fluid cells at gestational weeks 18–22^+6^.

## Results

### Patients and Genetic Background

**FIGURE 1:**
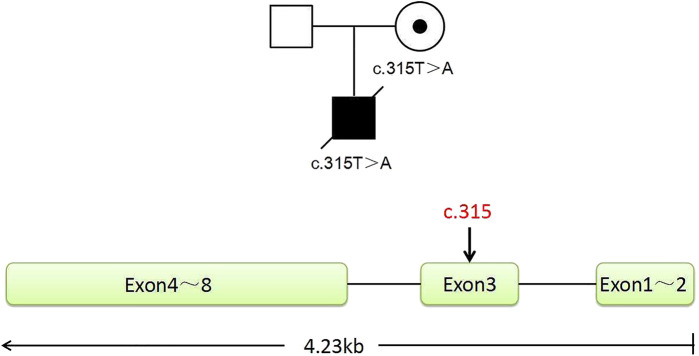
Pedigree of the family. The proband is a deceased hemizygous patient, and the woman is a disease-causing gene carrier, and the man is normal. The *IL2RG* gene with eight exons is located in the X chromosome Xq13.1. The graph shows the position of the mutant sites of c.315, located in exon 3.

In this family, this variant is the cause of X-linked SCID, whose hereditary mode is X-linked recessive inheritance (Figure 1). The size of the *IL2RG* gene is about 4.23 kb, containing eight exons (Figure 1). The c.315T > A variant occurred in exon 3. According to the ACMG/AMP guidelines, we evaluated this variant with PVS1 + PM2_Supporting as likely pathogenic. Both the husband and wife had normal karyotypes (320 bands). The peripheral blood of the couple and gDNA of the proband were detected by amplifying the target segments and Sanger sequencing (Supplementary Figure S1). Results showed that the female and proband were the carrier and hemizygote, respectively. The male did not carry this variant in the *IL2RG* gene (c.315T > A). These tests were carried out on the WGA production of BMCs from the carrier. The same diagnostic result was obtained as that of the peripheral blood cell test. The results indicate that the primers and MALBAC method could detect pathogenic variants in WGA products. (Supplementary Figure S1).

### TE Cell Biopsy and Detection of Pathogenic Variant Allele

**FIGURE 2:**
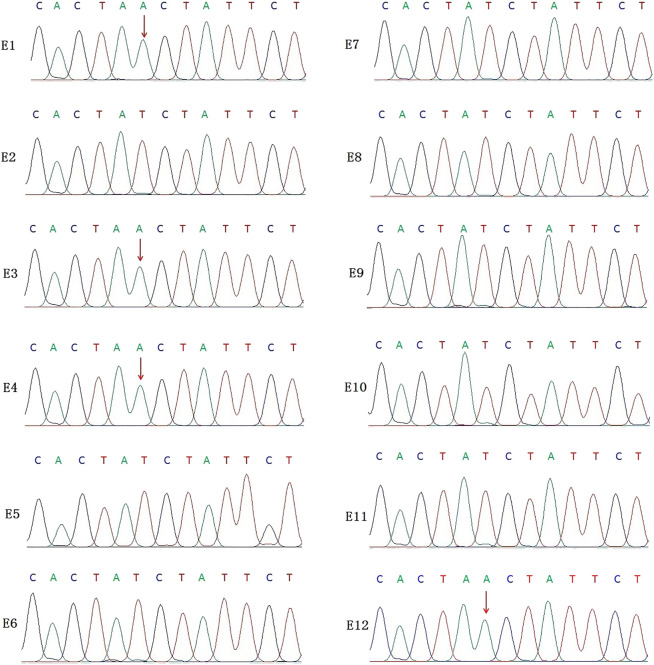
Results of the Sanger sequencing for biopsied TE cells. The figure shows that E1, E3, E4, and E12 are carriers of the *IL2RG* c.315 (T > A) variant; no variants were detected in the remaining embryo samples. For X-linked recessive diseases, this technique alone cannot distinguish between male patients and female carriers. At the same time, misdiagnosis lead by ADO cannot be ruled out.

In this PGT cycle, 12 embryos finally developed into blastocysts after ICSI (Table1). The TE biopsy was conducted by a mechanical method on day 5 or 6 post-insemination. A total of five to eight cells were collected and transferred into 0.2 μL PCR tubes, to which lysis buffer was added beforehand. The MALBAC two-step method was used for WGA. To detect the variant allele, parts of WGA products were purified to obtain 100 bp–10 kb fragments and eliminate primers, enzymes, and oligonucleotides of the previous reaction. Then, the purified products were detected by amplifying the target segments and Sanger sequencing (Figure 2). The data show that embryos E1, E3, E4, and E12 had a variant in the *IL2RG* gene c.315 (T > A). The remaining embryos either did not indicate variants or there was morbigenous ADO. Therefore, linkage analysis is necessary to determine if there is an ADO or chromosome recombination.

**TABLE 1:** Summary of detection results.

Biopsied Blastocysts	Gardner Grade	Copy Number Variations	SNP Haplotype	Sanger Sequencing
E1	4BC	—	male patient	c.315T > A
E2	4BB	46,XX	normal female	normal
E3	4BC	—	male patient	c.315T > A
E4	4BB	—	male patient	c.315T > A
E5	4BC	45,X,-X (×1)	abnormal detection	normal
E6	4BC	48,XX,+13 (×3),+16 (×3)	normal female	normal
E7	4B^−^C	46,XX, -Xq (q13.3→q28,∼80 Mb,×1,mos,∼50%)	normal female	normal
E8	4BC	46,XX, -4q (q34.3→q35.2,∼13 Mb,×1)	normal female	normal
E9	4BB	46,XX	normal female	normal
E10	4BC	46,XY	normal male	normal
E11	5BC	46,XX	normal female	normal
E12	4BC	—	male patient	c.315T > A

### SNP Haplotype

**FIGURE 3:**
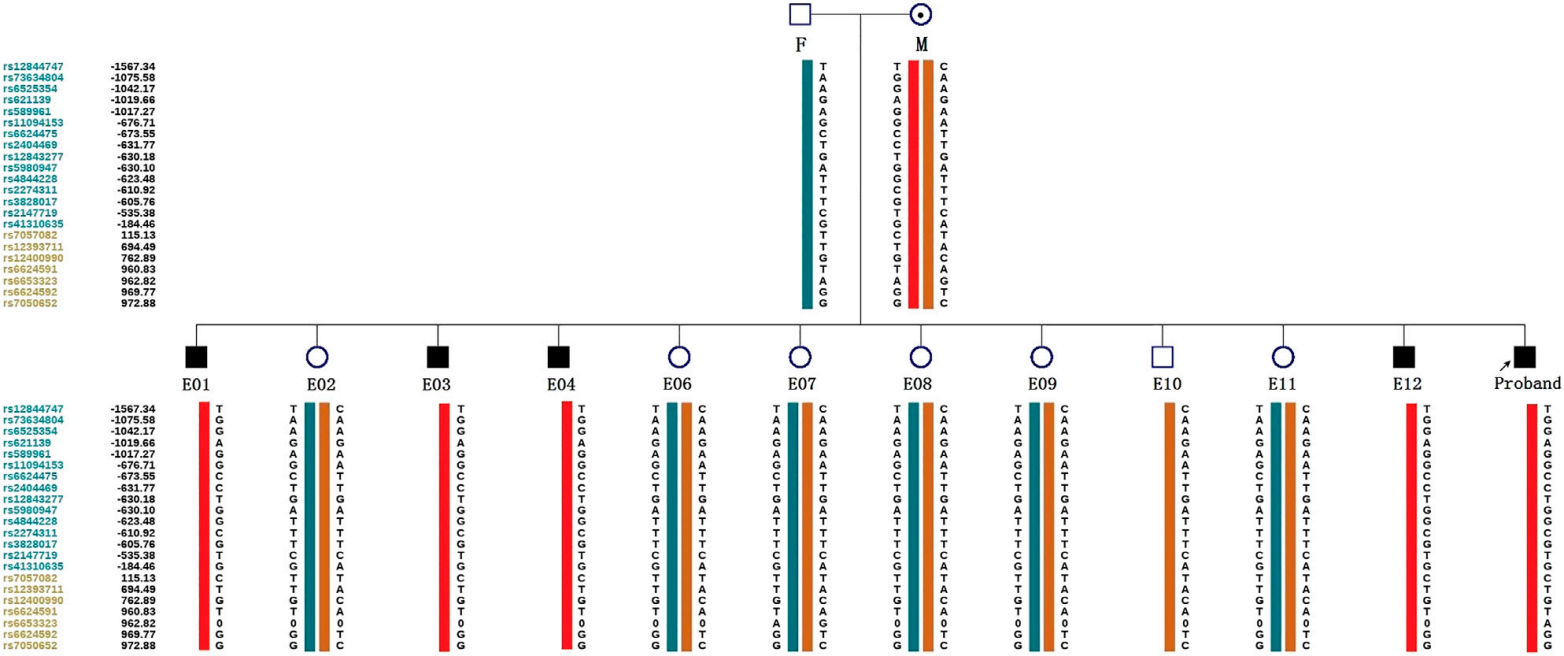
Schematic diagram representing the SNP-based haplotype of this family members and embryos. F, M, and P represent the father, mother, and proband, respectively. The reference SNP cluster ID numbers were listed on the left side. The ID numbers highlighted in dark blue and orange refer to the upstream and downstream informative SNPs, respectively. The red bar refer to the high-risk haplotypes, the dark blue bar represent the normal or low-risk haplotype of the father, and the dark orange bar represent the normal or low-risk haplotype of the mother. Results show that the E01, E03, E04, and E12 are male patient embryos; the E02, E06, E07, E08, E09, and E11 are normal female embryos; and the E10 is a normal male embryo. E05 was not included because of anomalies found in the linkage analysis.

ADO may lead to misdiagnosis. To minimize its interference with the diagnostic results, SNP markers within the 2 Mb region flanking the target gene were used for linkage analysis. The peripheral blood samples of this couple, gDNA of the proband, and WGA products of embryos were applied to the SNP haplotype (Figure 3). For the *IL2RG* gene, 60 SNPs were chosen for linkage analysis. In this case, within 1 Mb upstream and downstream of the variant site, there were 7 and 13 SNPs, respectively. The selection of informative SNP was based on the ESHRE PGT Consortium good practice recommendations for detecting monogenic disorders (Group et al., 2020). Combined with the pedigree haplotype, the high-risk maternal chromosome was indicated by a red bar (Figure 3); the dark blue and dark orange represent paternal and low-risk maternal chromosomes, respectively. Results show that E02, E06, E07, E08, E09, E10, and E11 did not carry the high-risk maternal chromosome. E05 is not listed in the graph because its linkage analysis cannot be determined, as it only had the paternal X chromosome; therefore, as we think it might be X monosomy, we will confirm our conjecture through PGT-A soon. The results of pathogenic variant site detection and linkage analysis were considered, and we can conclude that E02, E06, E07, E08, E09, E10, and E11 do not carry maternal c.315 (T > A) in the *IL2RG* gene.

### Preimplantation Genetic Testing for Aneuploidy (PGT-A)

The above seven embryos that did not carry the maternal variant were detected for aneuploidy. NGS-based CNV-Seq (CNV sequencing) was performed in the Illumina MiseqDx platform, indicating large than 10 Mb CNV and 30–70% mosaicism (large than 30 Mb). The CNV results are summarized in Table 1. No CNV larger than 10 M and aneuploidy were found in E02, E09, E10, and E11. E05, which could be determined for linkage analysis, indicated X monosomy (Supplementary Figure S2). E06 was aneuploidy, and E07 and E08 had fragment deletion or mosaicism (Supplementary Figure S2).

### Embryo Transplantation and Prenatal Genetic Diagnosis

For the embryo transplant decision, parents had discussions with a reproductive doctor, embryologist and medical genetic doctor in the Reproductive Medicine-Medical Genetic MDT (Multi-disciplinary Treatment, MDT) of the West China Second University Hospital. The decision was based on the developmental stage and grade of the embryo, according to PGT-M and PGT-A results (Table1). Finally, E02 was used for the transplantation, and the pregnancy was successful. At the 20th week of gestation, the pregnant woman underwent amniocentesis for prenatal genetic diagnosis. Amniotic cells were applied for CMA (cytoscan 750K, Affymetrix) (Lwvy and Wapner, 2018), amplification of the target segments, and Sanger sequencing (data not shown). The prenatal diagnosis was consistent with the preimplantation genetic testing. The woman eventually gave birth to a healthy girl who did not carry the variant.

## Discussion

SCID is an X-linked recessive monogenic hereditary disease characterized by defects in humoral and cellular immunity (Verbsky et al., 2012). Affected children often do not survive their first year of life due to recurrent and severe infections. PGT-M is an effective technique for blocking the transmission of this disease-causing gene. Until now, allogenic HSCT (Haddad and Hoenig, 2019) and gene therapy (Ferrua and Aiuti, 2017) are promising treatments for SCID (Suk See De Ravin, 2016). However, these two technologies still have limitations in clinical applications (Fischer and Hacein-Bey-Abina, 2020). For some inherited immunodeficiency like SCID, the PGT has become a practical technique for these at-risk couples to avoid affected pregnancies and have a healthy progeny free from genetic and chromosomal disorders (Rechitsky et al., 2018). At present, there is no mature gene therapy program for clinical application for children born with SCID, HSCT from a matched sibling donor is the most effective way. HLA matched stem cell transplantation improves significantly the outcome of transplantation treatment (Rechitsky et al., 2018). If children born with SCID are treated and protected early by pediatric ICU, they may be able to wait until PGT-M with HLA typing technique helps their family have a sibling who can provide a suitable transplantation donor.

We report a case blocking offspring with a rare inherited disease SCID by PGT-M. MALBAC is applied for WGA. An SNP-based haplotype and NGS were used for PGT-M&A by Illumina MiseqDx. Recently, MALBAC has been gradually recognized for its comparable single nucleotide variations detection efficiency, false-positive ratio, ADO ratio with MDA (Hou et al., 2015). Moreover, MALBAC is suited for the detection of CNVs because of its lower long-range variability in read mapping and weaker inherent bias in amplification than MDA (Charles F. A. de Bourcy, 2014).

Before the test protocol is applied to biopsied TE cells, we first detected gDNA of peripheral blood cells and WGA products of BMCs to evaluate the effectiveness and ADO rate of this method. Then we performed a PGT-M test on biopsied samples. For X-linked recessive inherited SCID, female carrier and normal embryo proceed to the next step of PGT-A. In linkage analysis, it was found that embryo 05 carried only the male normal X chromosome haplotype. In the Sanger sequencing of the pathogenic site, embryo 05 showed no variant. We hold the opinion that embryo 05 may be the X-monosomy inherited from the father; subsequent PGT-A results confirmed our conjecture. Therefore, to use PGT-M for X-linked genetic diseases, it is necessary to design a pair of primers to amplify the partial loci of the SRY gene or apply with PGT-A for embryo sex identification. For autosomal genetic diseases, PGT-A is also recommended after PGT-M. This helps to prevent us from misjudging the embryo in these cases. After PGT-M and PGT-A, E02, E09, E10, and E11 did not carry pathogenic variants, and no clear chromosomal abnormalities were found. E02 was eventually used for transplantation after considering the PGT results and morphological scores.

After a successful pregnancy by the PGT technique, prenatal diagnosis by amniocentesis during the second trimester is indispensable. Due to the possibility of mosaicism, biopsied trophectoderm cells represent only the genetic makeup of the placenta; there may be a normal placenta and abnormal fetal development. Therefore CMA and amplification of the target segments for Sanger sequencing were performed in amniocytes. This ensures that healthy babies are born through this double-check method.

In conclusion, PGT-M has important clinical application value in the prevention and control of birth defects. It can avoid the psychological and physical harm caused by induced labor after prenatal diagnosis. In this case, PGT (monogenic disorder and aneuploidy) combined with follow-up prenatal diagnosis helped a family with *IL2RG* gene variant have a healthy infant. We believe double detection at the blastocyst stage and second trimester, as well as the rational choice of multiple technologies can guarantee the birth of healthy children. At the same time, it also minimizes the risk of misdiagnosis caused by chromosome recombination, mosaicism, and ADO.

## Data Availability

The data analyzed in this study is subject to the following licenses/restrictions: The raw datasets analysed during the current study are not deposited in publicly available repositories because of considerations about the security of human genetic resources and patient anonymity, but are available from the corresponding author on reasonable request. Requests to access these datasets should be directed to Shanling Liu, sunny630@126.com.
