# Supplementary figures and images for: Case Report: Preimplantation Genetic Testing for X-Linked Severe Combined Immune Deficiency Caused by IL2RG Gene Variant
Source: Front Genet. 2022 Jun 1;13:926060. doi: 10.3389/fgene.2022.926060 (PMC9198258; doi:10.3389/fgene.2022.926060)

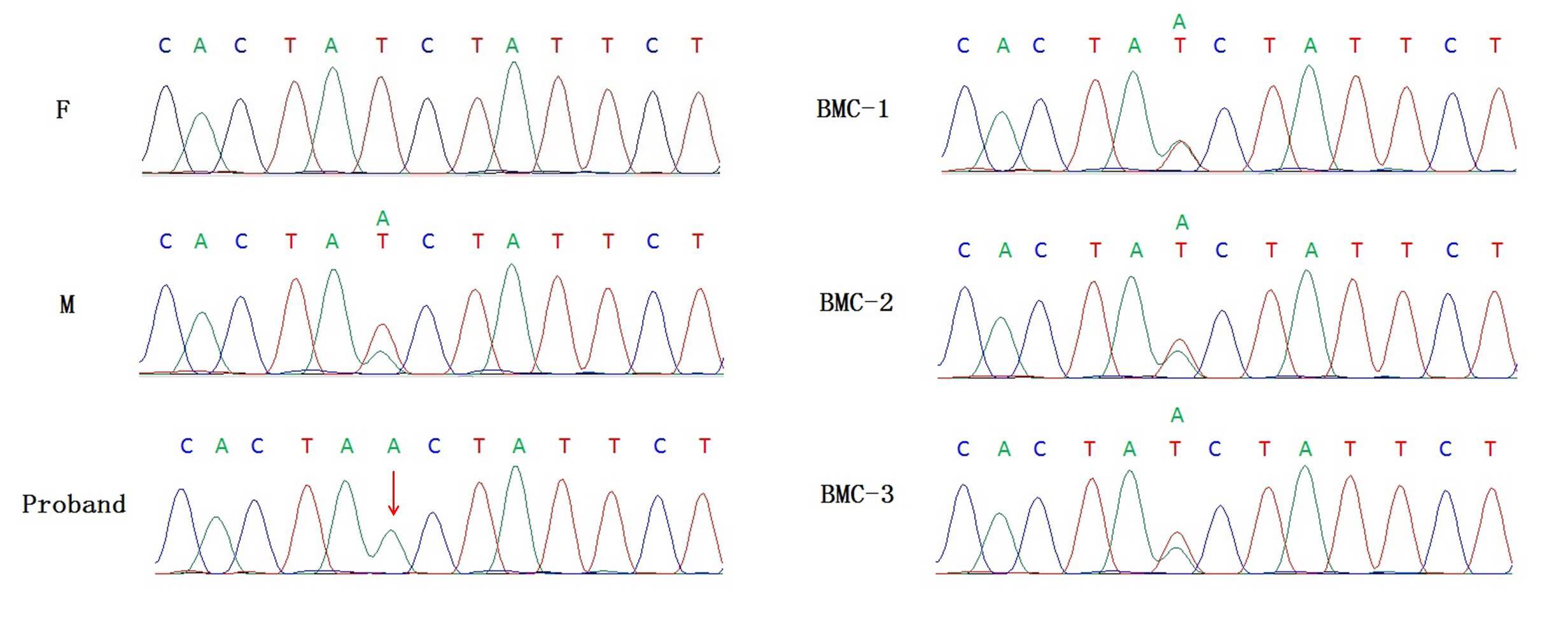

Supplement: Supplementary file 1 [file Image1.JPEG]

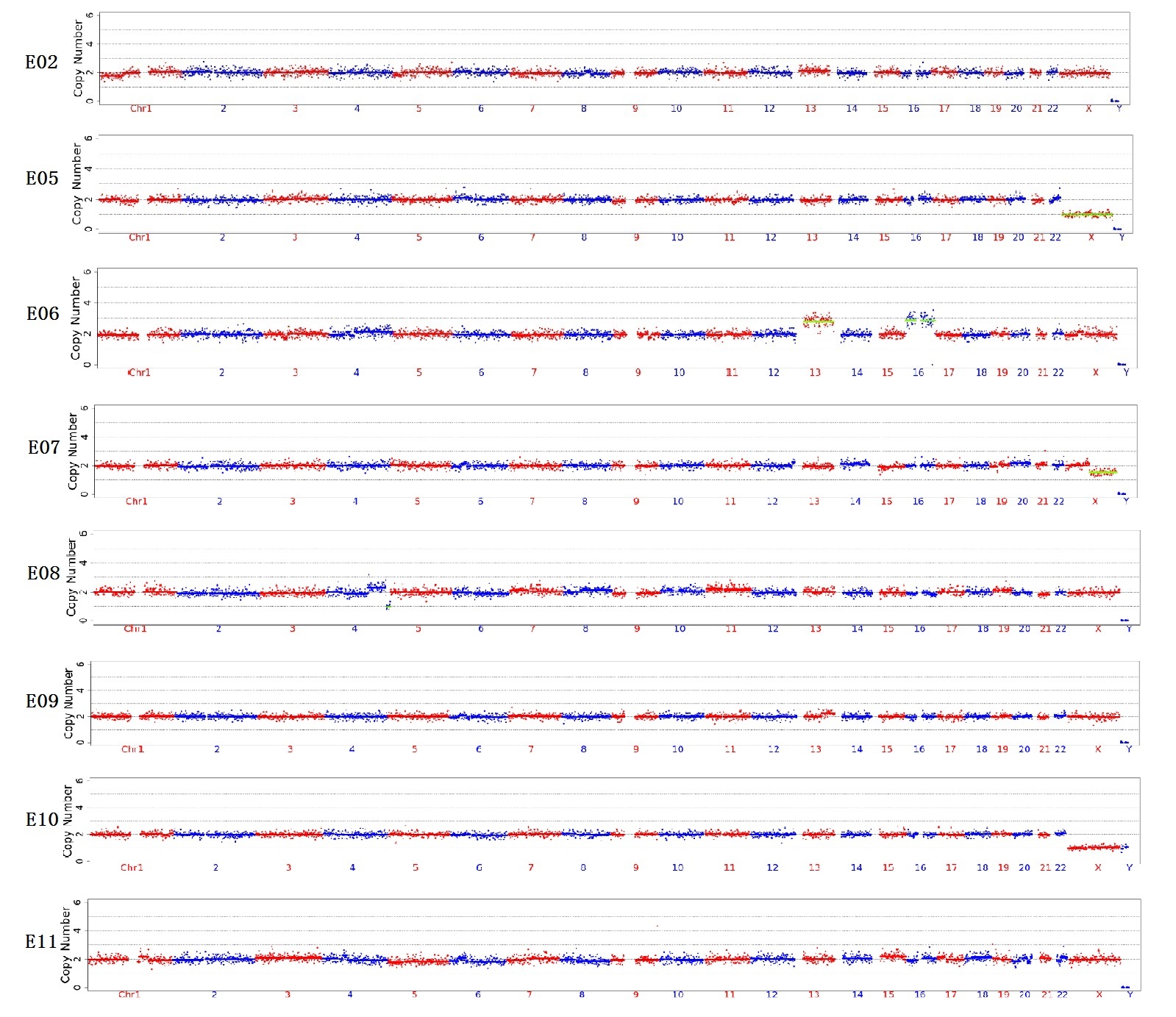

Supplement: Supplementary file 2 [file Image2.JPEG]
